# Development of an Onboard Robotic Platform for Embedded Programming Education

**DOI:** 10.3390/s21113916

**Published:** 2021-06-06

**Authors:** Hyun-Jae Lee, Hak Yi

**Affiliations:** 1 Keyon System, Namgu, Daegu 42415, Korea; jaelee6648@gmail.com; 2 School of Mechanical Engineering, Kyungpook National University, Bukgu, Daegu 42415, Korea

**Keywords:** robotics, embedded programming, engineering education, robotic platform, mechatronics

## Abstract

Robotics has been used as an attractive tool in diverse educational fields. A variety of robotic platforms have contributed to teaching practical embedded programming to engineering students at universities. However, most platforms only support content with a low level of programming skills and are unlikely to support a high level of embedded programming. This low association negatively affects students, such as incomprehension, decreased participation, dissatisfaction with course quality, etc. Therefore, this paper proposed a new robotic platform with relevant curricula to improve their effectiveness. The developed platform provided practical content used in mechatronics classes and the capability to operate a robot with a high level of embedded programming. To verify the effectiveness of the proposed platform, participants (undergraduates) examined course evaluations for educational programs based on the developed platform compared with the previous year’s class evaluation. The results showed that the proposed platform positively affects students’ intellectual ability (performance) and satisfaction in programming education.

## 1. Introduction

Robotics has been an attractive tool that offers high-performance capability in various educational institutions [1,2]. Above all, the use of robotic platforms has greatly contributed to providing a hands-on experience for engineering students at universities [3,4]. In addition, today’s latest technologies are being applied to robotics education to provide practical skills in a variety of fields of study [5,6].

The objective of early education in robotics is to provide students with opportunities to interact with robots. This can help students develop practical knowledge by solving project-based challenges with educational robots, as well as spark their interest in robotics [7,8]. Recently, as a further stage of learning robotics, it has progressed in the direction of inspiring students’ ingenuity beyond operating a simple robot to providing multidisciplinary education [9,10]. This shows that the shift in robotics education has a positive effect on the progress of learning high technology [11,12,13].

However, learning the conceptual design and basic programming skills applied to a robotic system remains a major part of robotics curricula at colleges [14]. Their uniform content implies a low connection with different subjects covered under the greater curriculum. This can result in a decrease in students’ participation, dissatisfaction with course quality, etc. In addition, except for using a complex and expensive platform, most robotics courses at universities have not clearly shown a distinct difference compared with those in K–12 education.

A new robotic platform with a detailed curriculum adapted for undergraduate students, including the core subjects of robotics, especially embedded programming, must be developed to address these issues [15]. Some research showed that robotics education using embedded programming has a positive impact on students [16,17], and this implies that students understand the application of programming languages and electrical electronics in robotic systems. Therefore, new robotics curricula must be developed to satisfy the problematic aspects of the current robotics classes.

This paper proposed a the new robotic system and robotics curriculum for undergraduate students to learn programming in robotics. Students participating in a class used the developed robotic platform to learn robotics fundamentals and focused on embedded programming. The proposed curriculum for a semester focused on operating robots using a high level of programming and can assist in solving a project-based challenge.

The rest of this paper is organized as follows. Section 2 explains the background of the education curriculum in robotics and robot competitions. Section 3 and Section 4 introduce the developed robot and the application of subjects based on robot development. Section 5 presents the results of the analysis examined by comparing student participation.

## 2. Background

The educational objective of using robots is to increase the practical knowledge and experience opportunities of the participants in robot operation.

**Figure 1:**
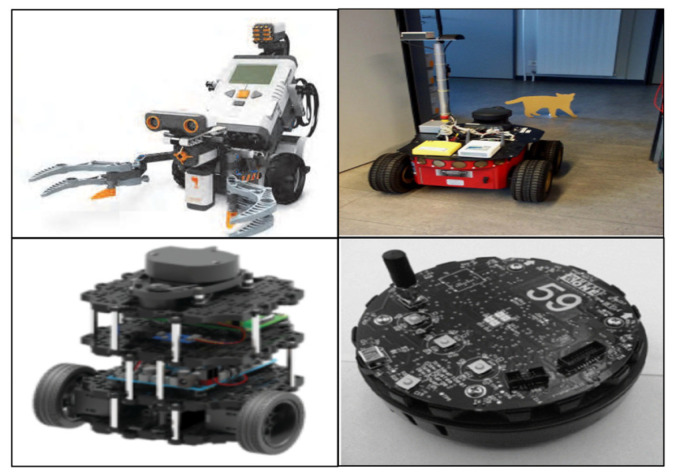
Educational robotic platform: LEGO kit, Pioneer, Turtlebot, and R-one platform.

Robotic platforms have contributed significantly to robotics education in various applications. LEGO Kit [3] and VEX Robotics’ Robot Kit [18], for which each component can be assembled, are effective platforms for realizing various mobile platforms that respond to their objective. Khepera [19], developed by K-Team, is a simple mobile platform (with two actuators and an infrared proximity sensor array) for controlling a robot with sensor communication. Pioneer [6] and Turtlebot [20], based on the Robot Operating System (ROS), are widely used to teach practical software programming. The R-one platform of Rice University [21] and Kilobot of Harvard [22] are swarm robotic platforms that are commonly used to teach the distributed control of multiple mobile platforms. Raspberry Pi- or Arduino-based robotics platforms are used to teach various practical robot applications effectively [23]. Figure 1 shows the educational robotic platforms used in robotics education.

**Figure 2:**
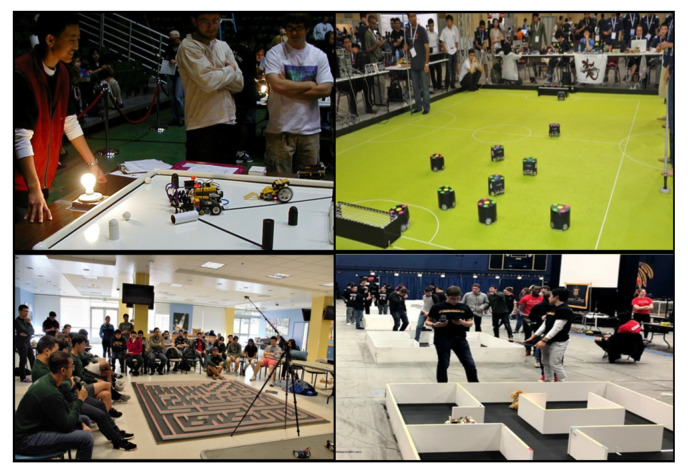
Robot competition: Botball, RoboCup Soccer, MicroMouse, and Firefighters.

Robot competition is also an effective program that attracts students’ active interest in robotics through their participation [24]. Among the robot competitions, MicroMouse is a tournament that develops autonomous driving algorithms for mobile robot to find an optimal path to solve a 16-by-16 size maze [25]. Firefighters is a competition in which many students compete to solve a mission by using a firefighting robot [26], and Botball uses a standardized kit to fabricate and operate a mobile robot [27]. RoboCup Soccer [28] has the highest degree of difficulty, which provides formidable challenges to advanced students. It has a positive effect on the student, such as enhanced problem-solving and cooperation abilities and increased interest in robots [29,30]. Robocon encourages students to practice their skills and analyze and solve the problem by creating robots with innovative technologies [31]. Figure 2 presents some robot competitions.

Programming education in robotics is also a powerful program for teaching programming skills by providing hands-on experiences through robotic platforms. Python programming on LEGO NXT, delta-robot, and UAV enables students to advance their programming skills [32,33]. Embedded programming using the LEGO NXT robot is an example of project-based learning used to improve students’ programming skills [34]. There is a C#-based programming course in operating a vision system applying robotic platforms (Roomba and LEGO NXT) to teach vision-based application [35]. ROS-based programming education applies the Gazebo simulator to teach both simulated and physical robots [36]. Furthermore, some STEM programming education uses Raspberry Pi or Linux computer to teach embedded systems [16,23].

However, most existing programs operate basic functions of educational robots, and few platforms have been developed to fully support a high level of embedded programming in undergraduate engineering education. Therefore, this study proposed a new onboard robotic platform with a relevant curriculum to provide the hands-on experience of operating robots through embedded programming.

## 3. Onboard Robotic Platform

This section introduces the hardware configuration, system architecture, and software tools of the developed robotic platform.

### 3.1. Hardware Configuration

**Figure 3:**
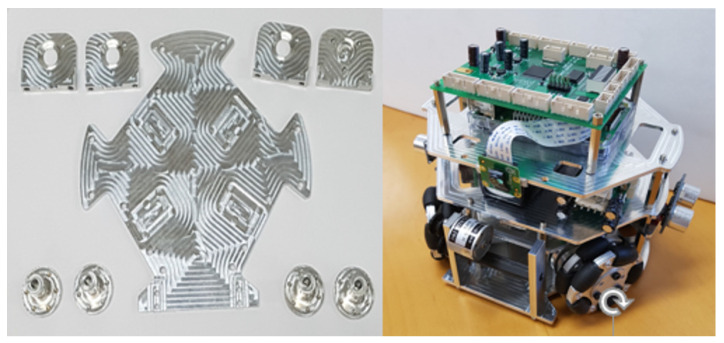
Mechatronics hardware configuration: component of body plate, the assembly of the onboard robotic platform.

The overall hardware configuration of the developed onboard robotic platform followed the RoboCup SSL competition’s rules. The specific configuration of the platform was considered for the project-based challenge to determine the sensors and actuators. The selected sensors and actuators were used to provide a practical embedded programming education. As shown in Figure 3, the developed platform had a 0.15 m height, a 0.18 m diameter, and a holonomic system with four omni-wheels. It was fabricated in Aluminum 6061 in-house; the total weight was 2.1 kg with three layers; the bottom layer included four BLDC motors (FL45BLW27) and a three-axis accelerator sensor (MAI-3AXIS-AN V2.0 module use LIS344ALH); the second layer consisted of ultrasonic sensors (HC-SR04), motor drivers, and a 24 V battery (six-cell LiPo battery with 2500 mAh); and the top layer had a vision module, a main controller, and a power distribution board. The maximum speed of the platform was 0.5 m/s, and the three-axis accelerator sensor was used to calculate the platform’s position. The ultrasonic sensor and vision module were used to detect obstacles.

**Figure 4:**
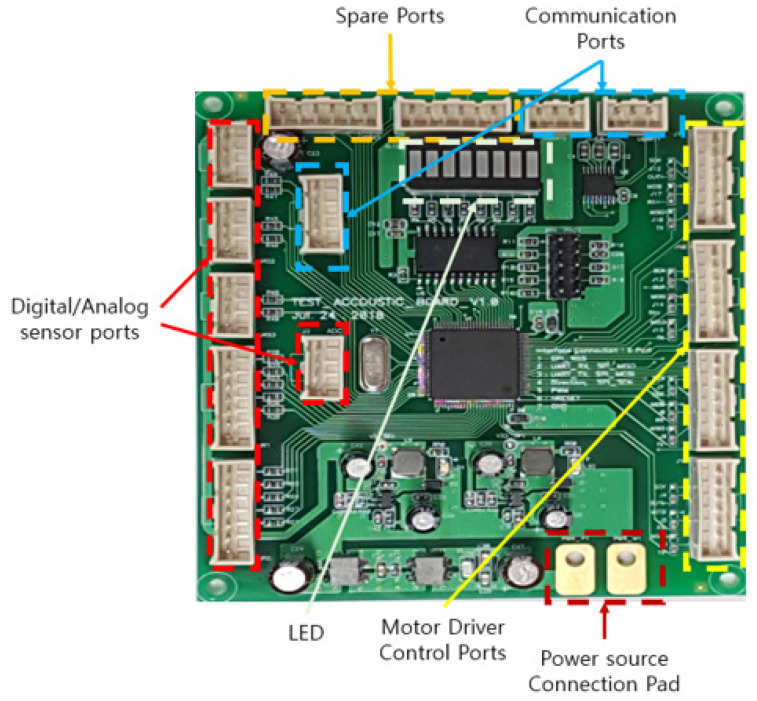
Configuration of the onboard robotic platform’s main controller.

Figure 4 shows the platform’s developed controller board driven by a 24 V source. The main controller included a source-distributing circuit that supplied voltage (3.3 V, 5 V) to the micro-controller (STM32777VIT6) and installed sensors. The STM32F family consists of high-performance micro-controllers that can control multiple sensors and actuators. Furthermore, it enables students to use the embedded programming environment more efficiently using the STM32CubeIDE software tool. The application of the main controller supports students in learning basic and high-level embedded programming. The main controller had four ports to drive and control the actuator module, detecting module (digital/analog sensors), communication module, vision module, and LED module. The communication ports had UART, RS232, and SPI communication pins, and the digital/analog ports consisted of GPIO and ADC pins. The motor driver control ports and spare ports had GPIO and timer pins. The detailed processes of controlling each module are explained in Section 5.3.

### 3.2. System Architecture

**Figure 5:**
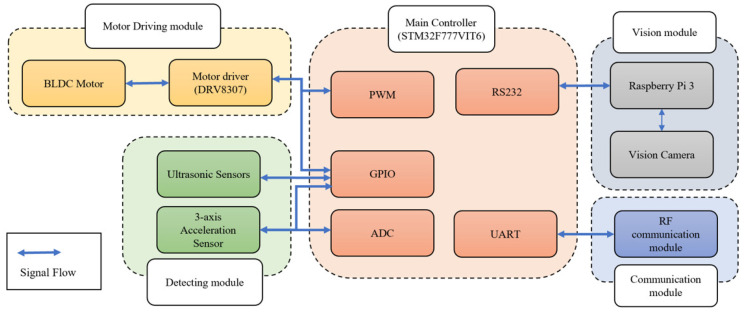
System architecture of the onboard robotic platform.

As shown in Figure 5, the STM32F micro-controller in the developed embedded system controlled each module using functions such as GPIO, ADC, PWM generation, and serial communication (RS232 and UART). The developed system consisted of four modules (motor driving, vision, detecting, and communication modules), and the details for each module are as follows.

**Figure 6:**
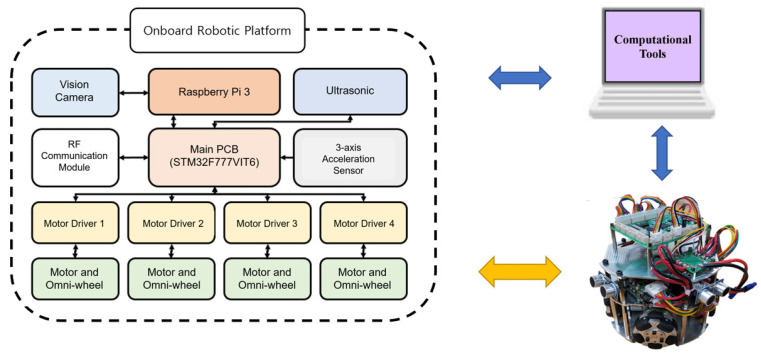
Entire communication architecture of the developed platform.

The motor driving module consisted of a motor driver and a BLDC motor. The motor driver receives PWM and GPIO signals from the main controller to control the speed and direction of the BLDC motor. The vision module consisted of a Raspberry Pi and a camera module. The camera module transmits vision data to the Raspberry Pi’s camera port, and the Raspberry Pi processes the vision data. The Raspberry Pi uses a serial communication port (RS232) to transmit processed vision data results to the main controller. The detecting module consisted of ultrasonic sensors and a three-axis acceleration sensor. It transmits data through the digital/analog ports. The main controller uses GPIO to obtain the object distance from the ultrasonic sensor, and the three-axis acceleration sensor uses GPIO and ADC to obtain the data. The communication module transmits and receives data with the other platform through wireless communication. The main controller uses the UART to transmit and receive data from the RF communication modules. Figure 6 shows the entire communication architecture of the developed platform.

### 3.3. Software

STM32CubeIDE, OpenCV, and MATLAB were used to control the developed platform. STM32Cube is an embedded system development software that provides pin settings for embedded system development environments. It helps manage actuators, communicate with external devices, and in the transmission/reception of sensor data. OpenCV was used to control the vision camera module and vision data-processing. The Raspberry Pi interfaces with serial communication to send processed vision data to the main controller. The main controller collects both vision and sensor data on the onboard robotic platform. Finally, a computational program (MATLAB) was used to monitor vision and sensor data received from the Raspberry Pi via the RF module or WiFi. MATLAB is one of the powerful computational programs in engineering education, and it provides a Simulink hardware support package. This package was used to support the onboard robotic platform to implement a monitoring system. Simulink receives calculated sensor data and applies them to a real-time platform monitoring system. The overall driving pseudo code for the onboard robotic platform is shown in Table 1.

## 4. Curriculum (Class Contents)

This section provides the curriculum associated with the onboard robotic platform, which consisted of basic and advanced levels, with the objective and the details of the class shown in Section 5. The basic and advanced levels focus on improving embedded programming skills using a micro-controller and merging the real-time monitoring system to execute a robot operation, respectively. Table 2 describes the program curriculum in detail.

The basic level provides fundamental and practical embedded programming and electrical electronics knowledge to users. The students first come to understand the system architecture of the platform and then learn the functions of the main controller (GPIO, timer/counter, interrupt, communication, and ADC). They also learn how to apply embedded programming with each module (driving, detecting, vision, and communication) of the mechatronics device through hands-on experience with the robotic platform and each module (driving, detecting, vision, and communication).

**Figure 7:**
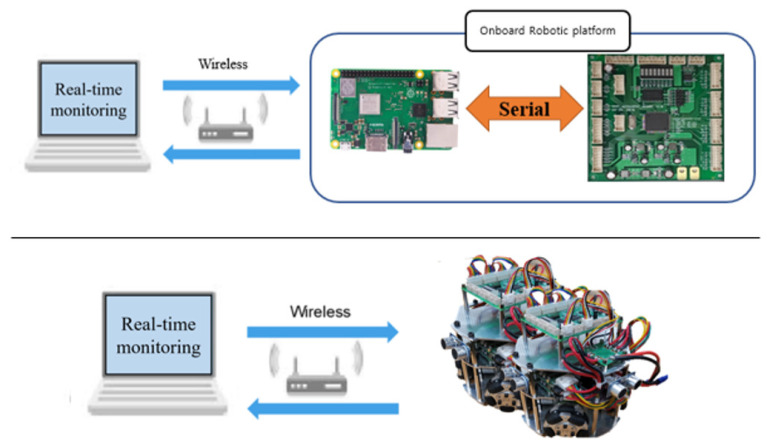
Implementation of the real-time monitoring system via the MATLAB Simulink hardware support package.

The advanced stage focuses on the operation of the developed robotic platform using programming skills. This stage consists of three steps: first, learning how to control the onboard robotic platform by integrating sensors and actuator modules; second, learning how to program using the hardware support package, as shown in Figure 7.

**Figure 8:**
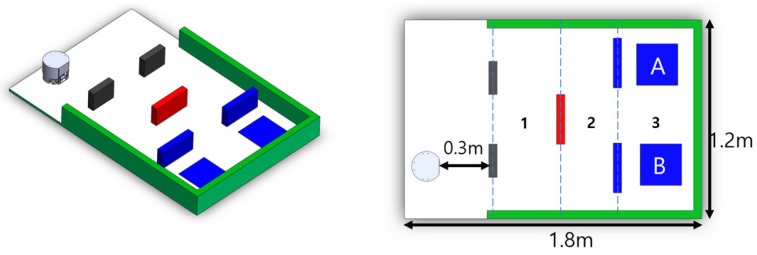
Driving field for the project-based challenge in the mechatronics class.

The last step is to program a project-based challenge to implement the platform’s autonomous driving through real-time platform monitoring. The driving environment consisted of three sections, as shown in Figure 8. The colors of the obstacles in each section were set differently. The ball position was randomly located at either Position A or B.

## 5. Participation Activity

This section describes the participation activity of undergraduate students and the effect of the proposed platform and curriculum.

### 5.1. Description

**Figure 9:**
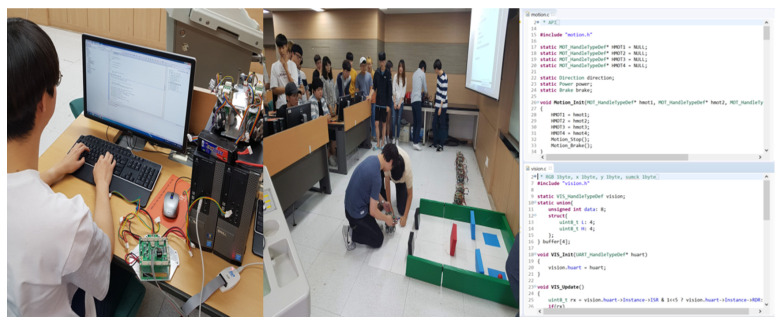
Participation activity in the Mechatronics class: (**left**) hands-on programming, (**middle**) project-based challenge, and (**right**) programming code.

The proposed platform and curriculum were used in a 300-level Mechatronics course for junior students at an engineering college. The participants were 40 junior engineering students, and the course was conducted for 16 weeks; each stage lasted eight weeks. The class met for 3 h per week, and students learned applicable practices such as system control of the main controller (STM32F777VIT6; Figure 9) using embedded programming and the operation of the robotic platform using a real-time monitoring system through the curriculum. The content of the curriculum is described in Table 3. The participants solved six tasks for 12 weeks, and the final project was conducted for the last four weeks.

Participants evaluated the developed platform with the relevant curriculum in two ways to verify its effectiveness. First, the questionnaires stated in Table 3 were used to assess the performance of the onboard robotic platform and the effectiveness of the curriculum. Q1 and Q2 were the questions to evaluate the satisfaction of the platform. Q3 and Q4 were the questions to determine the effects of the curriculum on programming education. Q5 and Q6 were questions that assessed the students’ intellectual ability. Each question was rated by the participants in points ranging from zero to ten from “strong disagreement(0)” to “strong agreement(10)”.

The second method was to evaluate the curriculum using student grades. Table 4 shows how student grades were rated by the assignment (T1 to T6) and the project-based challenge. The assignment consisted of four tasks (T1 to T4) related to the basic level of the curriculum and the others (T5, T6) for the advanced level. T1 and T2 were tasks for completing fundamental embedded programming through the main controller, and T3 and T4 were tasks for completing practical embedded programming through the application of the platform’s modules. T5, T6, and the project were tasks for completing the robot’s operation through high-level embedded programming.

The evaluation criteria for each task were as follows: perfect understanding and strong programming skills (8 to 10 points), perfect understanding, but weak programming skills (5 to 7 points), weak understanding and weak programming skill (3 to 4 points), and failure of achievement (0 to 2 points). The evaluation criteria for the project-based challenge were as follows: Successfully passing Section 1 (5 points), successfully passing pass Section 2 (8 points), and successfully passing Section 3 (12 points); Section 1 to Section 3 are also described in Figure 8. The scores were accumulated for each successfully passing section.

Furthermore, student evaluation results from the previous year’s 300-level Mechatronics course were used as a comparison to show the effectiveness of the developed platform with the relevant curriculum. The previous year’s Mechatronics course took the same amount of time (16 weeks), and the difference was that it did not apply the developed platform with the relevant curriculum. The list of the questionnaires, assignments, and project-based challenge in the previous curriculum was similar to those in Table 3 and Table 4, with the following differences: Q1 asked about satisfaction with the embedded programming kit; Q4 requested a practical integrating system; T3 employed diverse motors (DC, servo, and step motor) modules; T4 used the LCD module via serial communication; T5 was related to applying the vision module; T6 was integrating sensors and actuator modules; the project-based challenge was to design an imaginary system to solve a real-world engineering problem by implementing three functions including one novel function. The evaluation scores were based on the implementation of the functions: two functions (zero to seven points) and one novelty function (zero to eleven points); the total score was 25 points.

### 5.2. Results

**Figure 10:**
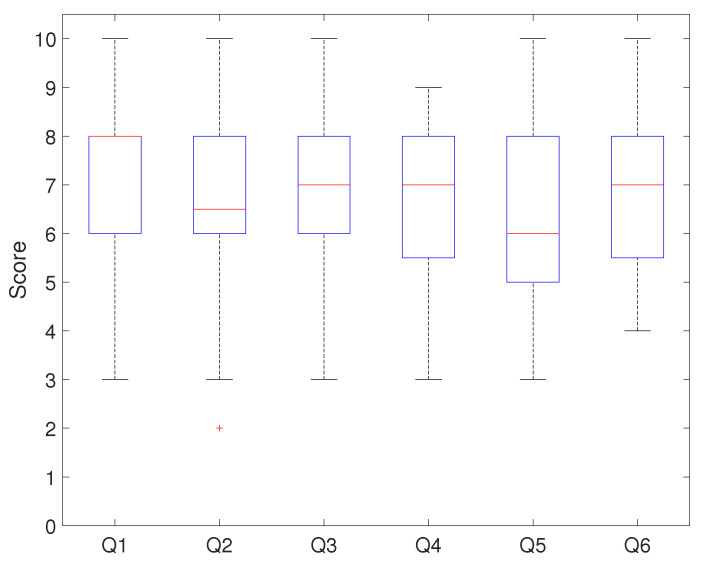
Points of each questionnaire question.

**Figure 11:**
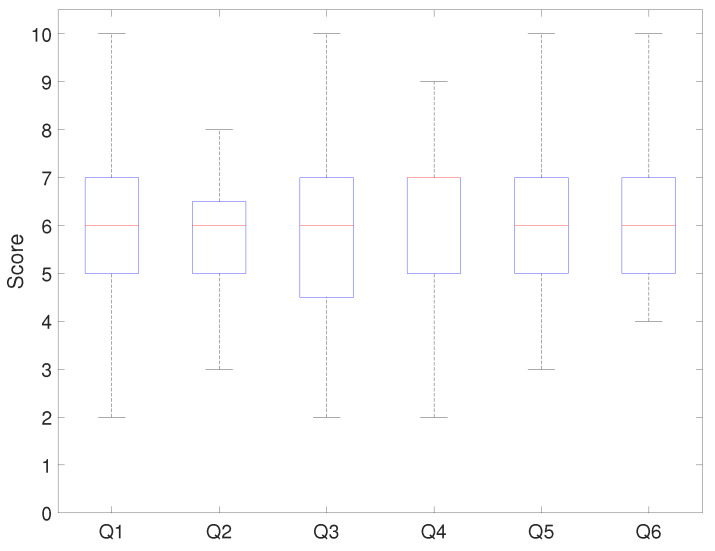
Points of each questionnaire question (last course).

Figure 10 and Figure 11 show the evaluation results of each questionnaire question. Figure 10 resulted from applying the developed platform with the relevant curriculum, and Figure 11 is the comparison result.

In Figure 10, the median score for Q1 was eight points, with 75% of students scoring above six. This showed that the students were satisfied with the hardware configuration. The median score for Q2 was 6.3 points, and seventy-five percent of students gave a positive review of the system architecture. When the score distribution for Q1 and Q2 was compared, the results showed that students were satisfied with the platform and system architecture. The median scores for Q3 and Q4 were the same (seven points), and seventy-five percent of students’ scores were above five. This showed that the developed onboard robotic platform with the relevant program had a positive effect on programming education. The median score for Q5 was six points, and approximately 75% of the students evaluated the curriculum as improving their mechatronics programming ability. The median score for Q6 was seven points, which showed that programming education positively affected their problem-solving ability. Figure 11 shows that the median score for Q1 was six points, with 50% of students scoring between five and seven points. The median score for Q2 and Q3 was six points, and fifty-percent of students evaluated this at around six points. The median score for Q4 was seven points, and seventy-five percent of students scored below eight points. The median score for Q5 and Q6 was six points.

**Figure 12:**
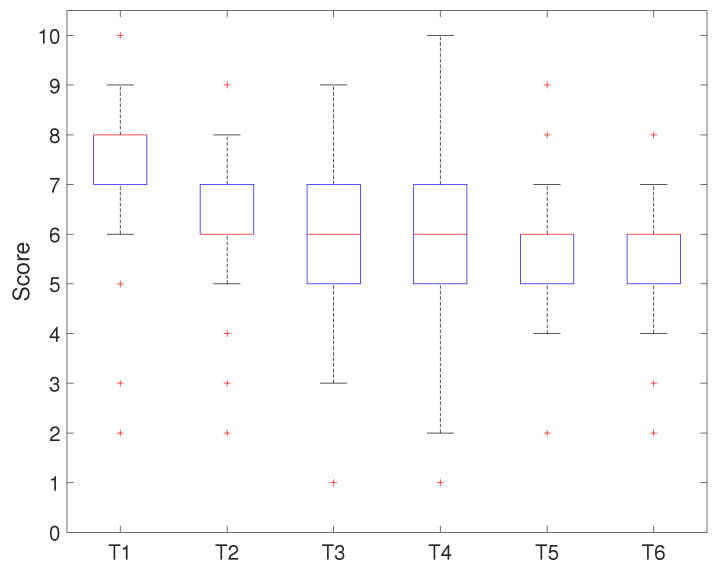
Scores of each assignment task.

**Figure 13:**
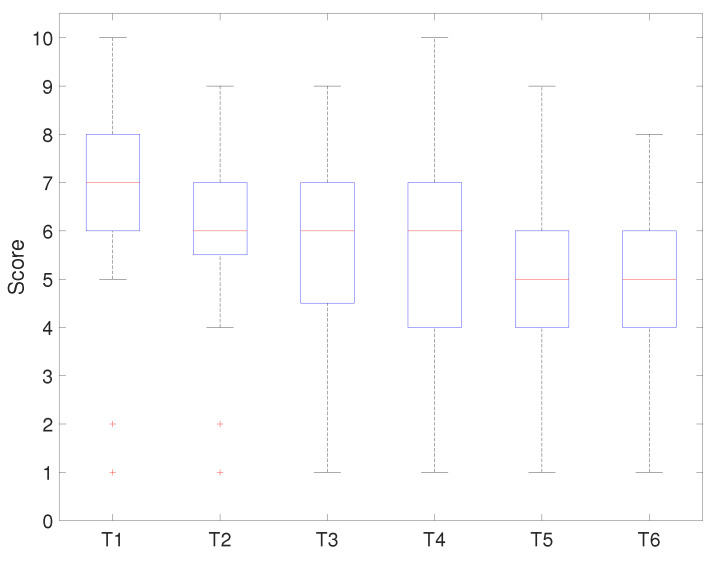
Scores of each assignment task (last course).

Figure 12 and Figure 13 show the evaluation result for each assignment task. Figure 12 shows the results of applying the developed platform with the relevant curriculum, while Figure 13 shows the comparison result.

Figure 12 shows that the median score for T1 was eight points, and seventy-five percent of students scored above seven points. The results showed that most of the students understood the basic concepts of GPIO, timer/counter, and interrupt and had strong programming skills. The result of T2 implied that the median score was six points, and seventy-five percent of students were above six points. This showed that most students understood the basic concept of communication and ADC, but they had weak programming skills. The results of T3 and T4 presented the same median score and a similar distribution. Seventy-five percent of the students’ outcomes were above five points, which showed that they understood the concept of the platform device’s modules, but they were very week at programming it. T5 and T6 showed similar results: the median score was approximately six points, and seventy-five percent of the students’ scores were below six points. This showed that the advanced stage-related tasks to drive the system were complex for the students.

Figure 13 shows that the median score for T1 was 7, and seventy-five percent of the students scored above six points. The median score for T2 was six points, and seventy-five percent of the students scored the same or below seven points. The results of T3 and T4 presented the same median score (six points) and a similar distribution. Seventy-five percent of the students scored less than seven points, which showed that most students understood the concept of the task, but they showed week programming skills. T5 and T6 presented similar results to the median score (five points) and score distribution.

**Figure 14:**
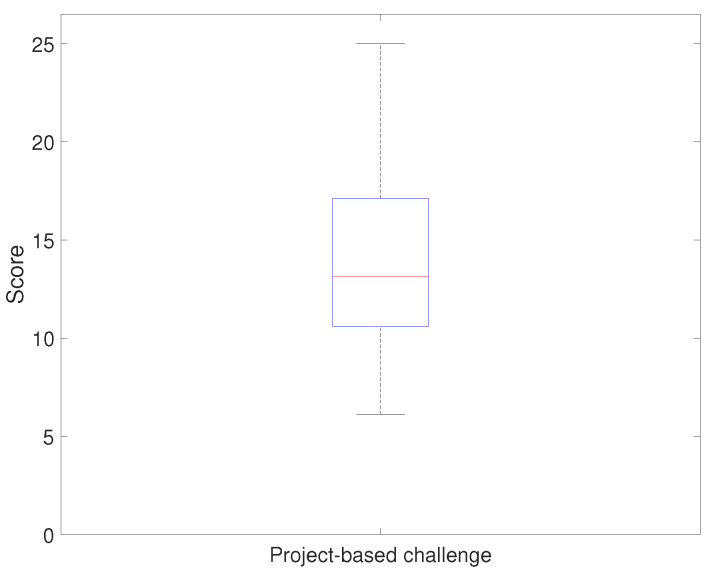
Evaluation results: final project (implementing an imaginary system to solve the real-world problem) scores (achievement evaluation).

**Figure 15:**
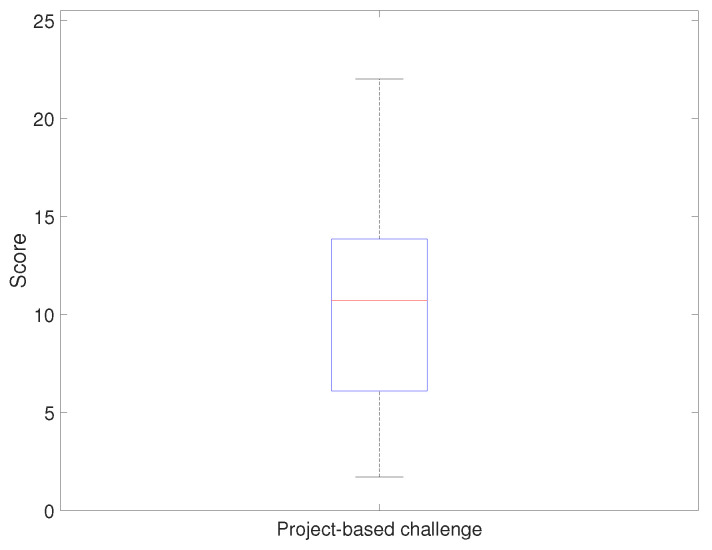
Evaluation results: final project (implementing imaginary system) scores (achievement evaluation).

Figure 14 and Figure 15 show the evaluation result of the project-based challenge. Figure 14 shows the results of applying the developed platform with the relevant curriculum, and Figure 15 shows the comparison result. The project-based challenge gave a a median score of 13 points and a minimum score of six points. According to the evaluation criteria, more than 50% of the students successfully passed at least two sections and implemented the driving environment using the real-time platform monitoring system. Furthermore, the minimum scores showed that all students successfully implemented at least one section of the device’s operating environment. The result of the project-based challenge in the previous course showed that the median was 10.7 points. The comparison result is discussed in the following subsection.

### 5.3. Discussion

The median score and score distribution of each showed that it improved compared to the previous course evaluation. In particular, Q1, Q2, and Q3 showed higher scores than the previous year’s evaluation scores. This implied that the developed platform with the relevant curriculum presented its effects through a positive review from the participants. Furthermore, the score for Q4 and Q5 showed a lower increase than the other scores, implying that the curriculum did not have a positive impact compared to the previous course. In summation, the overall median scores increased compared to last year’s course, but the response to both Q4 and Q5 showed that more content would be needed to improve students’ intellectual ability.

Students’ performance of each assignment task showed a similar tendency to decrease the score distributions as the task’s difficulty increased. Despite the similarity, the average score distribution of each task increased, showing that the new curriculum positively affected their performance. Furthermore, the basic level tasks’ score showed students ability to operate the system using embedded programming based on a high level of understanding. However, the results implied that students had difficulty operating the platform using practical embedded programming at the advanced stage. This showed that integrating modules and controlling complex system were difficult tasks for students to complete in a given period of time (two weeks).

The project-based challenge scores showed that the undergraduates successfully implemented a real-time monitoring system using a high level of embedded programming. Furthermore, they showed advanced performance compared to the previous curriculum project.

Therefore, the new robotic platform with the relevant curriculum improved students’ performance by increasing the content connection between practical robot operation and a high level of embedded programming. This also proved that it is an essential educational factor for future engineering education.

## 6. Conclusions

This study showed the newly developed onboard robotic platform with the relevant curriculum in the Mechatronics class applied. The results showed both the positive setup goals (achievement) and reviewed comments from participants (undergraduates). The application in the 300-level Mechatronics course implied that the developed platform with relevant courses provided satisfaction to the participants. Furthermore, it also contributed to the importance of the newly developed curriculum, which was effectively used in teaching practical embedded programming, which was not well covered in existing classes. Therefore, offering practical knowledge of operating robots using a high level of embedded programming to engineering students, it was very competitive. In future work, the curriculum will be further evaluated from additional courses in developing curriculum to improve theses results. Furthermore, qualitative statements will be collected to clearly identify difficulties that students encounter during the curriculum.

## Figures and Tables

**Table 1:** Pseudo code for the main controller and vision module in the communication processing of the vision module. T: transmit; R: receive.

4cPseudo Code for Main Controller and Vision Module			
**Procedure**	robotic platform	**Procedure**	vision module
	driving algorithm		algorithm
**Set Parameters**	Motor_speed[4],	**Set Parameters**	Vision data
	Motor_direction[4],		Communication
	A_sensor[8],		
	D_sensor[6],	While(1)	
	UART_pins,	{	
	RS232,	**Measurement**	Object detection
While(1)			(color and shape)
{		**Communication**	Serial comm.
**Measurement**	Object (distance, shape)		T: shape and color data
	Position and Speed		R: sensor data
**Decision**	Direction and speed		Wireless comm.
**Action**	Control actuators		T: sensor data
}		}	

**Table 2:** Detailed curriculum.

Level	Contents	Details
Basic	Fundamental function of the main controller (embedded programming)	Definition of the onboard robotic platform and system architecture Definition and application of GPIO
		Definition and application of both timer/counter and interrupt
		Definition and application of communication
		Definition and application of ADC
	Application of the sensors and actuator modules (practical embedded programming)	Application of the driving module (drive and control BLDC motor)
		Application of the detecting module (digital/analog sensors)
		Application of the vision module (Raspberry Pi + camera module)
		Communication module and basic programming
Advanced	Driving robotic platform and application of high-level embedded programming	Driving the onboard robotic platform (practical embedded programming)
		Application of programming (real-time platform monitoring system)
		Project-based challenge: operating an onboard robotic platform and real-time monitoring system

**Table 3:** List of questions in the questionnaire.

**Question**	**Contents**
Q1	Satisfaction with the hardware of the onboard robotic platform
Q2	Satisfaction with the system architecture of the platform
Q3	Effects on embedded programming learning
Q4	Effects on practical robot operation education
Q5	Development of intellectual ability
Q6	Improvement of problem solving ability

**Table 4:** Details of the assignment and project.

**Task**	**Contents**	**Goal of the Task**
T1	Control LED with GPIO, timer/counter, and interrupt	Understand functions of GPIO, timer/counter, and interrupt (via LED module)
T2	Communication between the PC and main controller applying ADC	Understand functions of communication and ADC, (via RS232)
T3	BLDC motor control based on digital/analog sensors	Understand the driving module and control BLDC motor (GPIO, timer/counter, and ADC)
T4	Communication between the main controller and vision module	Understand the vision module (Open CV, Raspberry Pi, and serial communication)
T5	Driving platform (embedded programming)	Integrate the platform’s modules to operate the robotic platform
T6	Platform monitoring system	Application of programming (via the platform monitoring system)
Project-based challenge	Application of the real-time robotic system	Small competition (application of Task 5 and Task 6)

## Data Availability

Not applicable.

## Abbreviations

The following abbreviations are used in this manuscript:

Omni-wheel	Omnidirectional-wheel
BLDC motor	Brushless DC motor
UART	Universal Asynchronous Receiver/Transmitter
RS232	Recommended Standard 232
SPI	Serial Peripheral Interface
GPIO	General Purpose Input/Output
ADC	Analog-to-Digital Converter
PWM	Pulse Width Modulation
PC	Personal Computer
Comm	Communication
